# New-onset seizure In Peripartum period: a case of Sjögren’s syndrome with renal artery stenosis

**DOI:** 10.1093/omcr/omaf199

**Published:** 2025-09-15

**Authors:** Rahul Parajuli, Santosh Basyal, Agnimshwor Dahal, Rebicca Pradhan

**Affiliations:** Intensive Care Unit, Hospital for Advanced Medicine and Surgery (HAMS), Mandikhatar Road, Kathmandu 44600, Nepal; Nepal Medical College and Teaching Hospital, Jorpati-Sundarijal Road, Gokarneshwor, Kathmandu 46600, Nepal; Nepal Intensive Care Research Foundation, Bansbari, Kathmandu 44600, Nepal; Internal Medicine, Hospital for Advanced Medicine and Surgery (HAMS), Mandikhatar Road, Kathmandu 44600 Nepal

**Keywords:** Sjögren syndrome, vasculitis, seizure, autoimmune, peri-partum period

## Abstract

Sjögren’s syndrome (SS) is an autoimmune disorder that results in chronic inflammatory and degenerative alterations in the exocrine glands and systemic organs, with an estimated incidence rate of 6.92 per 100 000 person-years. This case illustrates an uncommon correlation in a patient who developed new-onset seizures during the peripartum period, primary Sjögren’s disease (pSS), and chronic renal artery stenosis. pSS may go undetected when it manifests with other systemic disorders without classical symptoms. This paper emphasizes the distinct characteristics of pSS in pregnant women, proposing that the RAS, identified prior to the diagnosis of SS and new-onset seizure during this admission, may signify vasculitis consequence.

## Introduction

Primary Sjögren’s syndrome (pSS) is a systemic autoimmune disorder, with a 9:1 female-to-male ratio, typically peaking around the age of 50 [1]. Patients commonly experience fatigue, musculoskeletal pain, neurological symptoms and occasional extra-glandular manifestations including arthritis, Raynaud’s phenomenon, vasculitis, lymphadenopathy, serositis, pulmonary fibrosis, central nervous system (CNS) symptoms, and renal tubular acidosis (RTA) [2]. 12–27% of patients have renal impairment in pSS [3]. Neurologic involvement affects roughly 20% of individuals with pSS. Nevertheless, diagnosing SS with neurological involvement can be challenging, and CNS signs are infrequently reported [4]. Women with SS have more pregnancy difficulties than those without. Research on the pregnancy outcomes of women with SS remains limited which shows that it is more complicated, and such patients had poor fetal outcomes [11]. Here, we describe a case of primary SS in a 33-year-old pregnant female who had a history of bilateral renal artery stenosis (RAS) and new-onset seizure.

## Case presentation

A 33-year-old female, gravida 2, para 1, at 16 weeks of gestation, presented with two episodes of new-onset seizures, both acute, characterized by limb stiffening, tongue biting, and spontaneous resolution. Her significant medical history included RAS diagnosed 10–15 years prior managed with stent placed in the left renal artery which later became nonfunctional, leading to left kidney atrophy. Following the procedure, she was started on aspirin, statin, and oral antihypertensive medications. Furthermore, she was diagnosed with pSS three years ago, following symptoms of dizziness, on-and-off headache, dry mouth, and dry eyes. Workup revealed Schirmer’s test with 0 mm on bilateral eyes, low complement factor III, positive antinuclear antibody (ANA), and anti-Sjögren’s syndrome-related antigen A (Anti-SSA) 60 kDa and 52 kDa antibodies (Table 1). Initially treated with Hydroxychloroquine (HCQ), she discontinued it 7–8 months prior (Fig. 2) due to perceived worsening of sicca symptoms. HCQ was resumed 16 weeks into her current pregnancy during her first Antenatal checkup (ANC). Her first pregnancy, eight years prior, was uneventful delivered via lower segment cesarean section (LSCS) for breech presentation.

**Table 1:** Investigations (a) Blood serum (b) autoimmune panel (c) Immunological panel (d) Iron profile (e) radio imaging (f) CSF analysis.

Investigations		
Test name	Result	Normal range
**a. BLOOD REPORTS (SERUM)**		
WBC (Total count)	5410/mm^3^	4000–10 000/mm^3^
Absolute neutrophil count (ANC)	2.89	1.8–7.8
Reticulocyte %	2.52%	0.5–2%
Hemoglobin	8.7 g/dl	11–16 g/dl
Platelet count	80 000/mm^3^	150 000—400 000/mm^3^
PCV	27.6%	
Urea	14.4 mg/dl	
Creatinine	0.76 mg/dl	
Sodium	140 mmol/l	
POTASSIUM	3.8 mmol/l	
CALCIUM	9.7 MG/DL	
MAGNESIUM	2.3 MG/DL	
ESR	9 MM/HR	
CRP	NEGATIVE	
**b. AUTOIMMUNE PANEL INVESTIGATION**		
dsDNA ELISA	6.2 IU/ml (Negative)	
Sm/RNP	Negative (<6)	
SSA/Ro 60 kDa	86 (Positive)	
SSA/Ro 52 kDa	118 (Positive)	
Scl-70	Negative (<6)	
Jo-1	Negative (<6)	
ANA (ANF) ELISA Qualitative	Positive	
**c. IMMUNOLOGICAL INVESTIGATIONS**		
C3	65.20 mg/dl (Low)	75–135 mg/dl
C4	0.215 g/dl	0.1–0.4 g/dl
ANTI-TRANSGLUTAMINASE IGA	2.0 (NEGATIVE)	
Anti-phospholipid antibody	Negative	
**d. IRON PROFILE**		
Serum Iron	5.2 UMOL/L	
Ferritin	4.70PG/ML	
Ferritin	203.9 ng/ml	
TRANSFERRIN	3.9 G/L	
TIBC	82.40 UMOL/HR	
**e. RADIOIMAGINGS**		
Doppler Bilateral Lower Limb (ARTERIAL)	Monophasic flow in distal arteries of the right lower limb. Otherwise, normal arterial system of bilateral lower limb. No evidence of significant stenosis.	
Carotid doppler	Normal carotid duplex examination without hemodynamically significant flow disturbances. Normal vertebral-basilar system with antegrade flow	
CT Angiography of Abdominal Aorta	Significant stenosis abdominal aorta inferior to origin of right renal artery (approximately 50%) Left renal artery is seen to arise from the involved segment with hyperdense structure in situ, probably stent. (*Findings similar to previous scan dated 30/07/2015)*	
CT Angiography of Abdominal aorta	The infra-renal abdominal aorta shows mild luminal narrowing distal to origin of right renal artery for an approximate length of 3.5 cm with minimal transverse diameter of 7.5 * 6.5 cm. The pre-stenotic suprarenal aorta measures 14 * 12 mm and post-stenotic infra-renal aorta measures 14 * 13 cm. Rest of the aorta shows mild atherosclerotic changes. The left renal artery shows a patent stent in its proximal segment and small sized distal renal artery and its segmental and subsegmental branches. Left kidney is small and atrophic and measures 7.4 * 2.2 cm. Right kidney shows compensatory hypertrophy and measures 11.6 * 4.8 cm.	
**f. CSF ANALYSIS**		
Color and appearance	Watery, clear	
Sugar Protein Total count ADA	56 mg/dl 11.9 mg/dl Nil 0.5 U/l	

**Figure 1:**
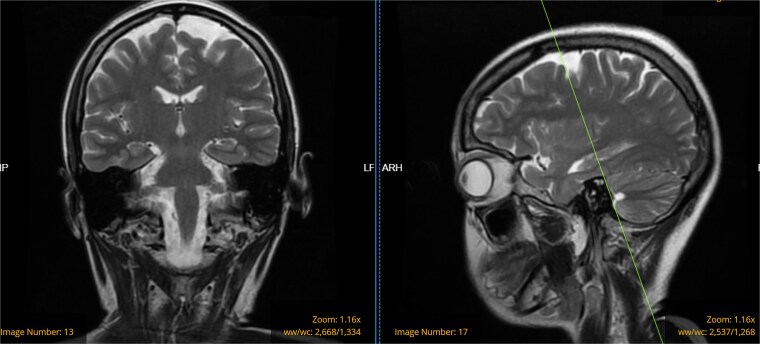
MRI T2 flair (a) axial and (b) coronal showing mild bi-frontal atrophy.

On this admission, the patient was afebrile, hemodynamically stable, and neurologically intact apart from the witnessed seizures. Neurological workup was done, which included magnetic resonance imaging (MRI) of the brain, which revealed mild bifrontal atrophy without acute pathology (Fig. 1). Lumbar puncture was performed, and cerebrospinal fluid (CSF) was sent for analysis, which was normal, ruling out CNS infections (Table 1). Obstetric ultrasonography (USG) was performed, which revealed a single live intrauterine fetus corresponding to 16 weeks and 2 days of gestation with cephalic presentation and normal fetal parameters.

The patient was admitted to the High Dependency Unit (HDU) for neuromonitoring and was managed with Inj. Levetiracetam for seizure control, HCQ continuation, and calcium and folate as pregnancy support. A multidisciplinary approach that included neurology, rheumatology, and obstetrics teams collaborated on care. She was kept under neuromonitoring for 24 hours, during which her vital signs were stable and seizures did not recur. Later, she was discharged with oral Levetiracetam to prevent further episodes and regular supplements for pregnancy support. She is on regular follow-up for ANC with an obstetrician, neurologist for new-onset seizure and rheumatologist for pSS. Both the fetus and the mother remain in optimal health during ANC and subsequent follow-ups.

We utilized validated study tools such as European League Against Rheumatism (EULAR) Sjögren’s Syndrome Patient Reported Index (ESSPRI) and EULAR Sjögren’s Syndrome Disease Activity Index (ESSDAI) to measure the severity and activity of pSS, respectively. ESSPRI: 6.66 (High disease activity: ⩾5, Low disease activity < 5) ESSDAI: Used to assess disease activity.

## Discussion and conclusion

We present a 33-year-old female during 16 weeks of gestation with pSS and chronic RAS who presented with a new-onset seizure. Along with sicca features, the patient tested positive for anti-SSA antibodies, ANA by an immunofluorescence assay, hypocomplementemia, and negative rheumatoid factor. Due to the lower sensitivity and the absence of SSB as a diagnostic criterion in the American College of Rheumatology (ACR)/EULAR classification [5], we did not utilize anti-La testing for the diagnosis of Sjögren’s syndrome.

In this case, the patient had bilateral RAS diagnosed 14 years ago. While fibromuscular dysplasia (FMD) causes RAS in young females, atherosclerosis is another primary etiology [6]. In our instance, RAS was induced by atherosclerosis. This may be attributable to vasculitis as a consequence of SS, which has resulted in atherosclerosis as the underlying cause of RAS. A computed tomography angiogram (CT-A) showed that the left renal artery was 99% stenosed. SS may have been present before the onset of RAS, but it was initially undiagnosed. During further assessment, the patient’s newly developed hypertension led to an incidental diagnosis of RAS. We hypothesize that SS may remain unrecognized when it presents along with other systemic disorders without classical symptoms.

Systemic autoimmune disorders impact various organ systems and engage both the central and peripheral nervous systems (PNS). Seizures represent a prevalent neurological manifestation and may occasionally serve as the initial symptom. The patient experienced tonic–clonic seizure, which is only seen in 3% of all SS patients [7]. While only six cases of SS associated cerebellar atrophy have been reported [8], the bi-frontal atrophy seen in our case (Fig. 1), appears novel.

Neonatal lupus and congenital heart block (CHB) are well-known fetal outcomes in pregnancies affected by SS [9], thus highlighting management considerations for the pregnant patient with pSS and RAS, including regular ANC and obstetrics scans. So, this case report also centers on a pregnant patient, emphasizing the importance of routine prenatal examinations to identify and prevent potential issues with the fetus and providing the patient with appropriate advice.

This case report highlights the diverse reno-vascular, neurological, ophthalmic, and dermatological manifestations in a pregnant patient with pSS. We believe the RAS, found incidentally preceding the diagnosis of Sjögren’s syndrome and mild bi-frontal atrophy might indicate a non-specific small and large vessel vasculitis complication associated with the syndrome itself.

**Figure 2:**
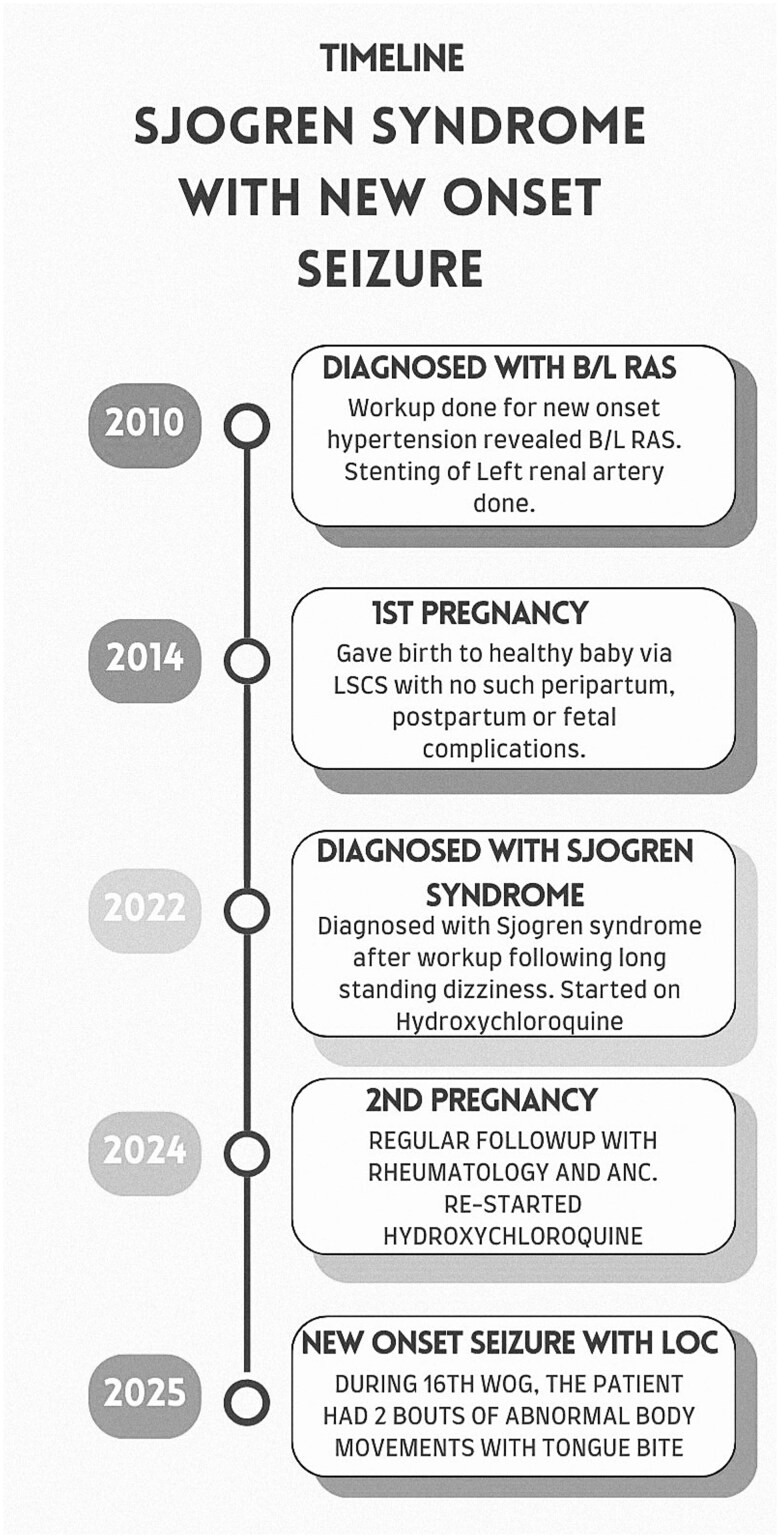
Chronology of events in a 33-year-old female with Sjögren’s syndrome with symptoms, diagnosis, comorbidities, therapy, and obstetric status.

Due to resource limitations, a vessel biopsy to definitively diagnose vasculitis was not obtained. Following careful evaluation, supported by symptoms and signs, investigations and diagnostic imaging, we initiated HCQ (Fig. 2). Follow-up showed significant improvement with no seizure recurrence and improved sicca symptoms.

Considering that no prior research reports such a complex presentation of SS in a pregnant patient, our findings are intriguing. Therefore, a multidisciplinary strategy is necessary for managing SS in the peri-partum period. This case underscores the importance of clinical vigilance for pSS manifestations, especially in resource-limited settings. Future research could further explore the connection between pSS, RAS, and these neurological findings in pregnancy.
